# Melatonin Overcomes Cancer Multidrug Resistance by Downregulating ABCB1 Expression and Modulating Mitochondrial Function

**DOI:** 10.1111/jpi.70096

**Published:** 2025-11-04

**Authors:** Alba López‐Rodríguez, Laura Martinez‐Ruiz, Raquel Morales‐Gallel, Javier Florido, Fabiola Bermejo‐Casares, José Manuel Garcia‐Verdugo, María Martín Estebané, Víctor Carriel, Noelia Maldonado‐Pérez, Pilar González‐García, Seyedeh T. Ahmadpour, Yolanda Ramírez‐Casas, Francisco Martín, Jean‐Francois Dumas, Christophe Vandier, Yang Yang, Darío Acuña‐Castroviejo, Germaine Escames

**Affiliations:** ^1^ Institute of Biotechnology, Biomedical Research Center, Health Sciences Technology Park, University of Granada Granada Spain; ^2^ Department of Physiology, Faculty of Medicine University of Granada Granada Spain; ^3^ Centro de Investigación Biomédica en Red Fragilidad y Envejecimiento Saludable (CIBERFES) Instituto de Investigación Biosanitaria (Ibs), Granada, San Cecilio University Hospital Granada Spain; ^4^ Cavanilles Institute of Biodiversity and Evolutionary Biology University of Valencia Valencia Spain; ^5^ Centro de Investigación Biomédica en Red sobre Enfermedades Neurodegenerativas (CIBERNED) Madrid Spain; ^6^ Department of Histology, Tissue Engineering Group, Faculty of Medicine University of Granada Granada Spain; ^7^ Instituto de Investigación Biosanitaria ibs. GRANADA Granada Spain; ^8^ Gene and Cell Therapy Unit, Genomic Medicine Department Pfizer‐University of Granada‐Junta de Andalucía, Centre for Genomics and Oncological Research (GENYO) Granada Spain; ^9^ Niche, Nutrition, Cancer and oxidative metabolism (N2Cox) UMR 1069, University of Tours, INSERM Tours France; ^10^ Key Laboratory of Resource Biology and Biotechnology in Western China Ministry of Education, Faculty of Life Sciences and Medicine, Northwest University Xi′an China

**Keywords:** apoptosis, head and neck cancer, melatonin, mitochondria, multidrug resistance, P‐glycoprotein, reactive oxygen species

## Abstract

Multidrug resistance (MDR) is a major challenge in cancer chemotherapy. A critical factor contributing to MDR is overexpression of ATP‐binding cassette (ABC) transporters, such as ABCB1. Novel alternative therapeutic strategies are needed to overcome resistance associated with ABC transporters. In the present study, we aimed to elucidate the mechanisms by which melatonin overcomes ABCB1‐mediated MDR in cancer cells, with a focus on mitochondrial function. We analyzed the effects of melatonin (1 mM) on head and neck squamous cell carcinoma cell lines (CAL 27 and SCC‐9) overexpressing *ABCB1* and exhibiting increased resistance to cisplatin (CDDP) compared to their parental cells. To further validate the role of melatonin in reversing ABCB1‐mediated MDR, we also evaluated its effects on doxorubicin‐resistant MCF‐7 breast cancer cells. We further examined the potential of melatonin to overcome MDR in CAL 27 xenografted mice. Here, we report that melatonin treatment specifically triggered reactive oxygen species (ROS) production in mitochondria and weakened chemoresistance. ROS oxidized NADH into NAD^+^, and limiting the availability of ATP for efflux pump activity. Additionally, melatonin decreased the number of mitochondria localized near the nucleus instead of the cytoplasm and downregulated ABCB1 expression. Intratumoral administration of melatonin effectively overcame CDDP resistance in CAL 27/ABCB1 xenografts, significantly reducing tumor volume and promoting apoptosis. These findings demonstrate that melatonin enhances chemosensitivity in ABCB1‐overexpressing cells by modulating mitochondrial metabolism, redox balance, and ABCB1 expression, highlighting its potential as an adjuvant therapy to overcome MDR.

## 1 Introduction

Clinical resistance to chemotherapeutic drugs remains an obstacle in the treatment of cancer patients [1, 2]. Multiple mechanisms have been identified in acquired chemoresistance [3, 4], and overexpression of ATP‐binding cassette (ABC) transporters has emerged as pivotal in multidrug resistance (MDR) [5, 6, 7]. These transmembrane proteins act as efflux pumps, actively transporting anticancer drugs out of the cell and reducing their intracellular concentration and efficiency. ABCB1 (P‐glycoprotein, P‐gp) is implicated in chemoresistance in a broad spectrum of malignancies [8, 9, 10], and its expression in different tumors has been associated with poor prognosis and outcomes [11, 12]. ABC transporters thus are considered important targets for reversing chemoresistance [13]. Therapeutic strategies aimed at overcoming MDR through inhibition of ABCB1 and other efflux transporters have had limited clinical success, however, because of insufficient specificity and adverse effects on normal tissue function [14].

ABC transporters exhibit a high ATP dependence [15], using ATP hydrolysis to fuel drug efflux from the cell, and recent studies have revealed relevant metabolic plasticity of mitochondria during chemotherapy [16, 17]. This metabolic shift likely compensates for the increased ATP demand of the efflux system [18, 19]. Moreover, redox homeostasis plays a critical role in MDR by mitigating chemotherapy‐induced apoptosis [7, 20]. Reduced intracellular accumulation of reactive oxygen species (ROS) signifies enhanced detoxification and suppression of apoptosis [21, 22, 23]. Through these mechanisms, mitochondrial and redox alterations in drug‐resistant cancer cells represent targets for overcoming resistance. Limitations such as potential toxicity in healthy organs and difficulty in specifically targeting mitochondria restrict the therapeutic window for inhibiting mitochondrial metabolism in cancer cells, however [7].

In this context, melatonin is a potent free radical scavenger that reduces apoptosis in normal cells [10, 11]. However, we previously reported that, in head and neck squamous cell carcinoma (HNSCC), melatonin enhances mitochondrial function and promotes apoptosis by increasing mitochondrial ROS (mtROS) through mitochondrial reverse electron transport [24, 25, 26, 27]. Despite increased mitochondrial activity, ATP production remains low, suggesting partial uncoupling between respiration and ATP synthesis [25, 28]. Of note, melatonin has been proposed to reduce tumor cell resistance to cytotoxic drugs by potentially modulating P‐gp expression or function, although data on this specific interaction are lacking. The purpose of this study was to examine pathways underlying the effects of melatonin in blunting the mitochondrial/ABCB1 connection and impeding the development of chemoresistance. The findings reveal new molecular mechanisms of melatonin in reversing chemoresistance and highlight a potentially effective treatment for tumor cells overexpressing ABCB1.

## 2 Materials and Methods

### 2.1 Cell Culture and Treatment

The HNSCC lines CAL 27 (ATCC: CRL‐2095) and SCC‐9 (ATCC: CRL‐1629) and the breast cancer cell line MCF7 (ECACC: CB‐2705) were obtained from the Cell Bank of the Scientific Instrumentation Centre of the University of Granada. Adriamycin‐resistant (ADR) MCF‐7 cells were obtained from the University of Tours [29]. Details are available in the supplementary information.

### 2.2 Lentiviral Vector Production

Lentiviral vectors were produced using HEK‐293T cells (ATCC). Cells were seeded in 10‐cm plates and transfected with a second‐generation three‐plasmid system comprising pLV[Exp]‐EF1A > hABCB1 (Vector Builder) as the vector plasmid, pCMVΔR8.9 (Addgene) as the HIV packaging plasmid, and pMD2.G (Addgene) as the VSV‐G envelope plasmid. Polyethylenimine (408727, Sigma, St Louis, MO, USA) served as the transfection reagent. Viral supernatants were harvested at 30, 48, and 72 h posttransfection, filtered through 0.45‐µm filters, and analyzed by flow cytometry using a Becton Dickinson FACSCanto II cytometer.

### 2.3 Proliferation Assay

Cell proliferation was assessed using the CyQUANT Cell Proliferation Assay Kit (C7026, ThermoFisher, Waltham, MA, USA) following the manufacturer′s instructions.

### 2.4 Measurement of CDDP Accumulation

Measurement of intracellular CDDP accumulation was performed using inductively coupled plasma–mass spectrometry (ICP‐MS) as previously described [30].

### 2.5 Western Blot Analysis

Proteins were extracted and analyzed by western blot analysis following previously described protocols [25]. Additional details can be found in the supplementary information.

### 2.6 Measurement of Doxorubicin Accumulation

After being treated with 3 µM doxorubicin and incubated at 37°C for 3 h, cells were washed and incubated at 37°C for 9 h without doxorubicin. Fluorescence was measured using a Lionheart FX automated microscope (Agilent Technologies, Santa Clara, CA, USA).

### 2.7 Measurement of Mitochondrial Respiration

The oxygen consumption rate was measured using the Seahorse XF‐24 Extracellular Flux Analyzer, following previously established methods [24]. Further details are provided in the supplementary information.

### 2.8 Electron Transport Chain Complex Activity Assays

The enzymatic activities of electron transport chain complexes were evaluated using commercial assay kits from Abcam, in accordance with the manufacturer′s instructions. Additional information is provided in the supporting materials.

### 2.9 NADH/NAD Quantification

Levels of NAD⁺ and NADH were measured using the NAD/NADH Assay Kit (ab65348, Abcam, Cambridge, UK) following the manufacturer′s instructions. Absorbance at 450 nm was recorded with a Power Wave X‐1 microplate spectrophotometer. Concentrations were calculated based on a standard curve and normalized to protein content determined by the Bradford assay.

### 2.10 Determination of ATP Levels

ATP concentrations were determined using the colorimetric ATP Assay Kit (ab83355, Abcam) in accordance with the manufacturer′s guidelines. Additional details are provided in the supplementary information.

### 2.11 Measurement of ROS Production and Mitochondrial Mass

Mitochondrial superoxide levels were assessed using the fluorescent probe MitoSox Red, while mitochondrial mass was measured with MitoTracker Green. ROS production was analyzed using the 2′,7′‐dichlorofluorescein diacetate (DCFDA) method, as previously described [25]. Further methodological details are provided in the supplementary information.

### 2.12 Apoptosis

Apoptosis was evaluated using an Annexin V/Propidium Iodide (PI) detection kit (ANXVKF‐100 T; Immunostep, Salamanca, Spain). After treatment, cells were stained with Annexin V and PI following the manufacturer′s instructions and subsequently analyzed by flow cytometry on a Becton Dickinson FACSCanto II system.

### 2.13 Cell Viability Assay

Cell viability was assessed using the Alamar Blue assay (DAL1025, Thermo Fisher Scientific, USA) following the manufacturer´s instructions. Cells were seeded at a density of 10.000 cells/well and were pretreated with the mitochondrial targeted antioxidant MitoTEMPO (SML0737, Sigma‐Aldrich, USA) at a final concentration of 100 µM during 1 h at 37°C, before exposure to the experimental treatments described in this study. After treatment, Alamar Blue reagent was added directly to the culture medium at a final concentration of 10% (v/v), and cells were incubated for 2 h at 37°C. Absorbance was measured at 570 nm using a microplate reader (Power Wave X‐1). Cell viability was expressed as a percentage relative to untreated control cells.

### 2.14 Cell Cycle Analysis

Cellular DNA content was measured by flow cytometry using a PI/RNase staining kit (Immunostep), in accordance with the manufacturer′s instructions. Samples were analyzed on a Becton Dickinson FACSCanto II cytometer, and results were reported as the percentage of cells in each cell cycle phase.

### 2.15 Animal Model and Treatments

Athymic nude mice (nu/nu), aged 5–6 weeks (Janvier Labs, France), were used to generate xenograft models from cancer cell lines. All animal procedures were conducted in accordance with ethical standards approved by the Institutional Animal Care and Use Committee of the University of Granada (protocol 15/10/2020/119), and complied with European (CETS No. 123) and Spanish (R.D. 53/2013) regulations. Additional details are provided in the supplementary information.

### 2.16 Magnetic Resonance Imaging (MRI)

Images were acquired using a Philips Achieva 3.0 TX MRI scanner fitted with an 8‐channel SENSE wrist coil. Further information is provided in the supporting materials.

### 2.17 Transmission Electron Microscopy

Microscopy image quantification was carried out using ImageJ software (version 1.54j; National Institutes of Health). Additional methodological details are provided in the supplementary information.

### 2.18 Histology

Hematoxylin–eosin staining was used to evaluate general tumor morphology. Collagen fiber distribution was assessed via Picrosirius red staining, while Alcian blue (pH 2.5) staining was applied to detect proteoglycans and mucopolysaccharides. Proliferative tumor cells were identified by immunohistochemical staining for Ki‐67 (MAD‐000310QD, Vitro SA, Madrid, Spain), and apoptotic cells were detected using the TUNEL assay (G3250, Promega, Madison, WI, USA), both following the manufacturers′ protocols. Image analysis was conducted using ImageJ software (version 1.54j; National Institutes of Health, Bethesda, MD, USA). Further details are available in the supplementary information.

### 2.19 Data and Statistical Analysis

Statistical analysis was conducted using Prism 8 software (GraphPad Software Inc.). Comparisons between experimental groups and their corresponding untreated controls were made using unpaired Student′s t‐tests. Data are presented as mean ± standard error of the mean (SEM) from at least three independent experiments. A *p*‐value < 0.05 was considered indicative of statistical significance.

## 3 Results

### 3.1 Verification of Chemoresistance With ABCB1 Overexpression and of Melatonin Cytotoxicity

**Figure 1:**
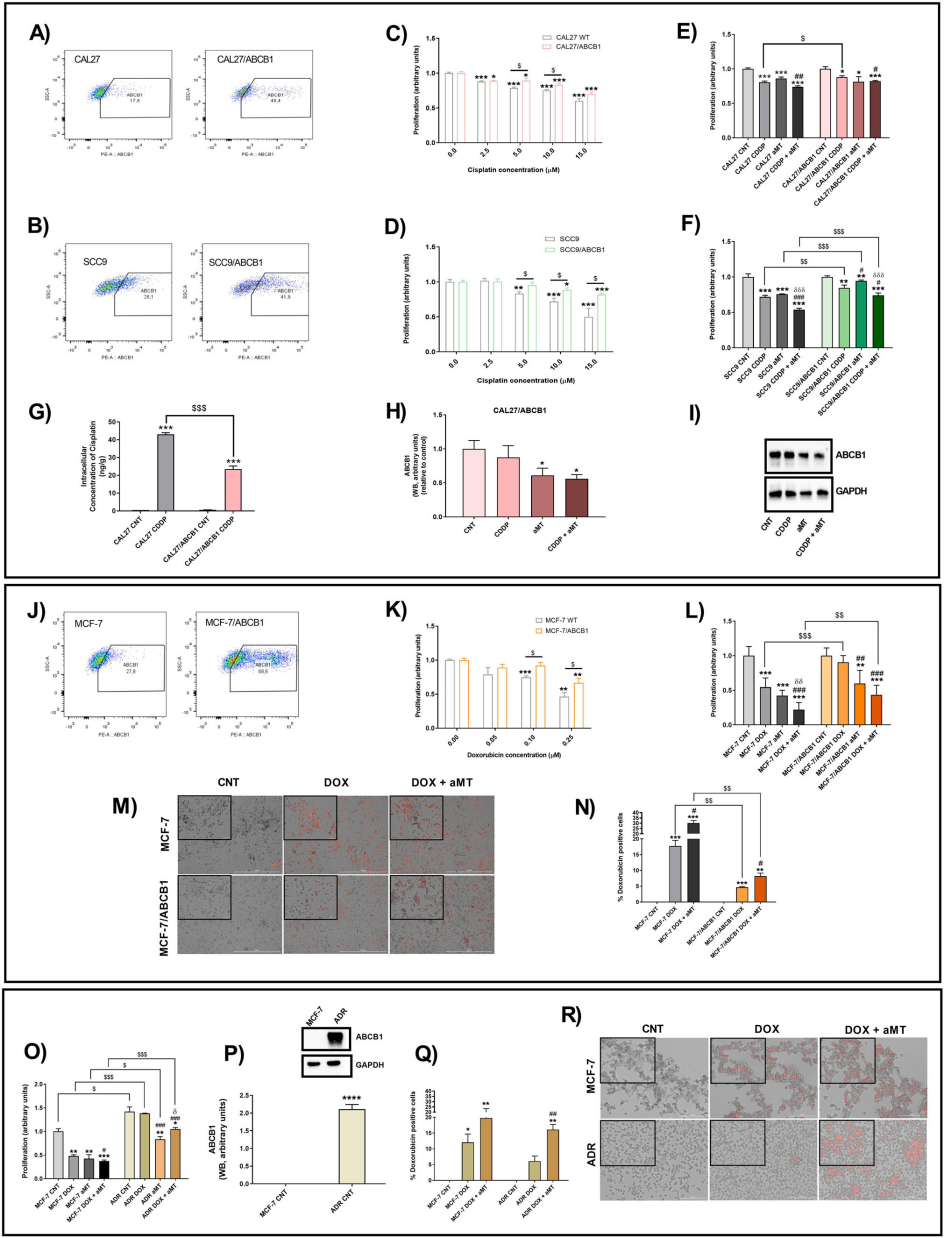
Melatonin restores the sensitivity of drug‐resistant cells to cisplatin and doxorubicin by inhibiting ABCB1 expression. (A, B) Analysis by flow cytometry of a lentiviral vector expressing the *ABCB1* gene in CAL 27 and SCC9 cell lines. (C, D) Proliferation rate after treatment with increasing concentrations of cisplatin in HNSCC‐ABCB1 and parental cells. (E, F) Proliferation rate after treatment with vehicle (control, CNT), melatonin (aMT) at 1000 µM for 42 h, cisplatin (CDDP) at 5 µM for 18 h, or combined treatment. (G) Intracellular concentration of cisplatin analyzed by ICP‐MS. (H, I) ABCB1 expression analyzed by western blot. Treatment groups included vehicle, aMT at 1000 µM for 42 h, CDDP at 5 µM for 18 h, or combined treatment. (J) Analysis by flow cytometry of a lentiviral vector expressing *ABCB1* in the MCF7 cell line. (K) Proliferation rate after treatment with increasing concentrations of doxorubicin (DOX) in MCF7‐ABCB1 and parental cells. (L) Proliferation rate after treatment with vehicle, aMT at 1000 µM for 42 h, DOX at 0.1 µM for 18 h, or combined treatment. (M, N) DOX accumulation by fluorescence microscopy in MCF7/ABCB1 and parental cells treated with DOX at 3 µM for 3 h and then incubated 9 h without DOX. (O) Proliferation rate after treatment with vehicle (control, CNT), aMT 1000 µM for 19 h, and DOX 3 µM for 5 h followed by 1 h incubation in drug‐free medium, or combined treatment in parental and ADR MCF7 cells. (P) ABCB1 protein expression in parental and ADR MCF7 cells, as determined by western blot. (Q, R) Fluorescence microscopy analysis of DOX accumulation in parental and ADR MCF7 cells treated with aMT 1000 µM for 19 h and DOX 3 µM for 5 h followed by 1 h incubation in drug‐free medium. WB, western blot. Data are presented as mean ± standard error of the mean (*n* = 3–12) for each group; one‐tailed unpaired t‐test; *p < 0.05; **p < 0.01; ***p < 0.001 versus control; ^#^ *p* < 0.05; ^##^ *p* < 0.01; ^###^ *p* < 0.001 versus CDDP or DOX group; ^δ^ *p* < 0.05; ^δδ^ *p* < 0.01; ^δδδ^ *p* < 0.001 versus aMT group; ^$^ *p* < 0.05; ^$$^ *p* < 0.01; ^$$$^ *p* < 0.001.

To assess melatonin′s effect on ABCB1‐mediated MDR, HNSCC cells were transduced with ABCB1 and analyzed by flow cytometry, confirming increased ABCB1 expression (Figure 1A,B). We first tested the cytotoxicity of CDDP in parental HNSCCs (CAL 27 and SCC9) and in the ABCB1‐overexpressing CAL 27/ABCB1 (Figure 1C) and SCC9/ABCB1 cells (Figure 1D). ABCB1‐overexpressing cells showed greater resistance to CDDP compared to parental cells, with significant differences at 5 μM CDDP (Figure 1C,D). This dose was used in all subsequent experiments.

Consistent with previous dose–response studies, cells were treated with 1000 µM melatonin, the concentration showing a maximal effect [24, 26, 31]. Co‐treatment with melatonin effectively reversed CDDP resistance in ABCB1‐overexpressing cells (Figure 1E,F; Table S1), restoring sensitivity to levels comparable to parental cells. Treatment with CDDP led to proliferation reductions of 19.4% in CAL 27% and 28.2% in SCC9 cells, whereas the decreases were only 12% in CAL 27/ABCB1% and 15.5% in SCC9/ABCB1 cells. When melatonin was combined with CDDP, melatonin enhanced CDDP cytotoxicity, and proliferation decreased to 18% in CAL 27/ABCB1% and 25.9% in SCC9/ABCB1 cells. Effectively, ICP‐MS analysis confirmed that ABCB1 overexpression decreased intracellular CDDP two fold (Figure 1G), whereas melatonin reduced P‐gp expression (Figure 1H,I), both alone and in combination with CDDP, confirming its role in reversing drug efflux.

To further validate these findings, we analyzed MCF‐7 and MCF‐7/ABCB1 breast cancer cells (Figure 1J). Unlike parental MCF‐7 cells, MCF‐7/ABCB1 cells were resistant to doxorubicin at concentrations below 0.25 µM (Figure 1K). With 0.1 μM doxorubicin, proliferation declined by 45.6% in MCF7 and by 9.5% in MCF‐7/ABCB1 cells (Figure 1L; Table S1). Melatonin significantly enhanced the cytotoxicity of doxorubicin in MCF‐7/ABCB1 cells, further decreasing proliferation compared to parental controls by 56.5% (Figure 1L; Table S1). We also analyzed doxorubicin accumulation (Figure 1M,N), and fluorescence microscopy showed significantly reduced doxorubicin concentration in MCF‐7/ABCB1 cells. However, melatonin co‐treatment increased doxorubicin accumulation in resistant cells, supporting its role in reversing ABCB1‐mediated chemoresistance.

Similarly, in ADR/MCF‐7 cells (Figure 1O–R), which also overexpress ABCB1 (Figure 1P), melatonin enhanced doxorubicin cytotoxicity, leading to a further decrease in cell proliferation (Figure 1O) by increasing its intracellular concentration (Figure 1Q,R), thereby overcoming drug resistance. These results suggested that melatonin may act as an ABCB1 inhibitor.

### 3.2 Melatonin Modulates Mitochondrial Metabolism in ABCB1‐overexpressing Cells

**Figure 2:**
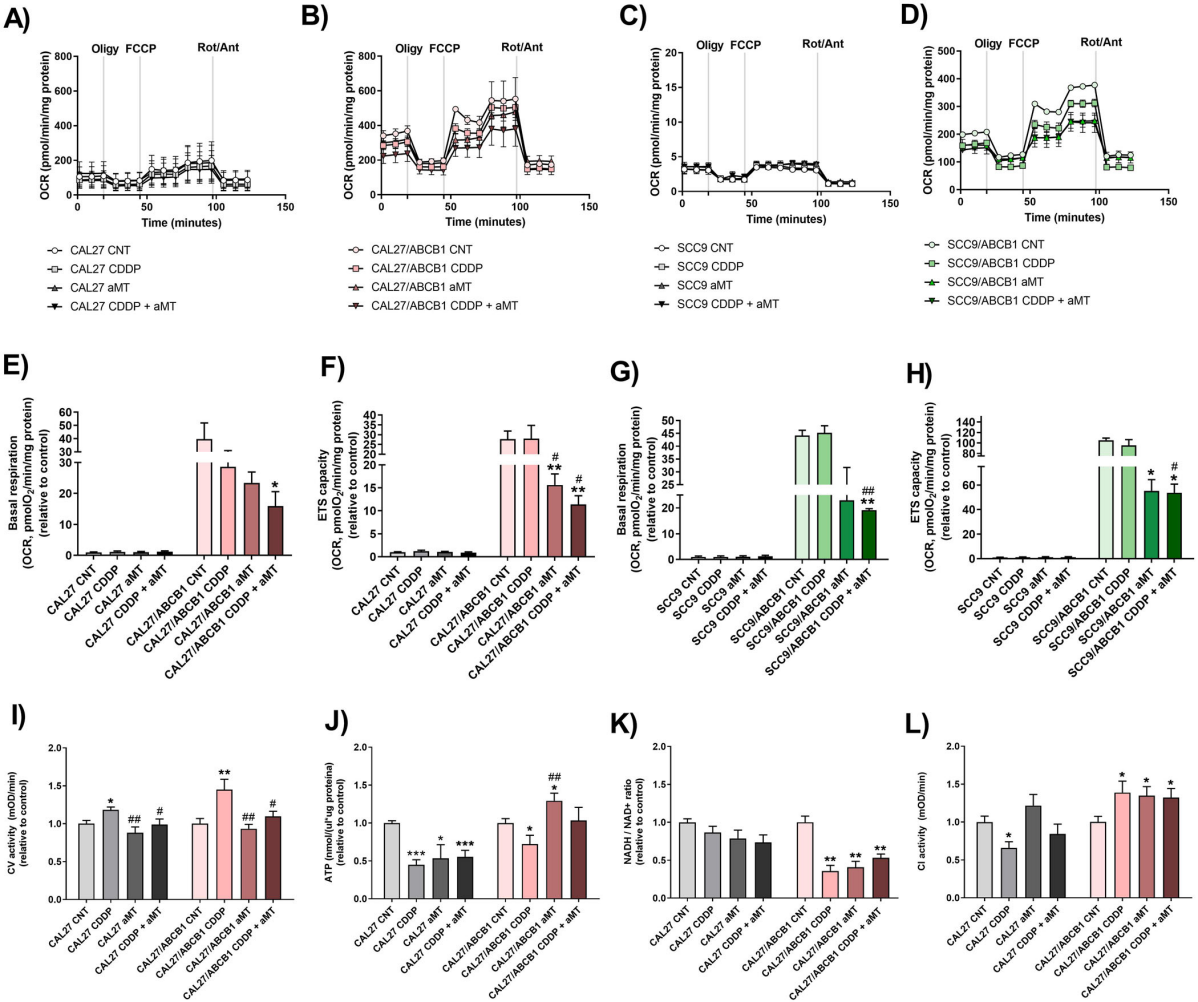
Melatonin regulates mitochondrial energy metabolism in HNSCC/ABCB1 cells. (A–D) Oxygen consumption rate corresponding to (E, G) basal respiration and (F, H) electron transport system capacity, as analyzed by SeaHorse in CAL 27 and SCC9 ABCB1‐overexpressing and parental cells. (I) Analysis of mitochondrial Complex V activity by spectrophotometric analysis. (J) ATP levels measured by fluorometry. (K) NADH and NAD+ levels measured using a colorimetric test and expressed as a ratio. (L) Analysis of mitochondrial Complex I activity by spectrophotometric analysis. Treatments include vehicle (control, CNT), melatonin (aMT) at 1000 µM for 42 h, cisplatin (CDDP) at 5 µM for 18 h, or combined treatment. Data are presented as mean ± standard error of the mean (*n* = 4–7) for each group; one‐tailed unpaired t‐test; *p < 0.05; **p < 0.01; ***p < 0.001 versus control; ^#^ *p* < 0.05; ^##^ *p* < 0.01 versus CDDP group.

Given the link between drug resistance and increased mitochondrial metabolism [16, 18], we analyzed mitochondrial function in HNSCC/ABCB1 and parental cells to explore melatonin′s role in enhancing chemosensitivity. We examined mitochondrial respiration using a SeaHorse XF24 extracellular flux analyzer (Figure 2A–D). CAL 27/ABCB1 and SCC9/ABCB1 cells exhibited higher basal and maximal respiration compared to parental cells (Figure 2A–H). Melatonin alone or in combination with CDDP significantly reduced electron transport system (ETS) capacity in MDR cells compared to controls and to the CDDP‐treated group (Figure 2F,H).

Given that elevated mitochondrial respiration supports ATP demands in ABCB1‐overexpressing cells (Figure S1A, B) and that inhibiting ATP production may help overcome drug resistance, we examined the effects of melatonin and CDDP on Complex V activity and ATP levels (Figure 2I, J). CDDP elevated Complex V activity, especially in ABCB1‐overexpressing cells (Figure 2I), likely because of increased ATP demand for drug efflux. Despite this, ATP levels decreased (Figure 2J), indicating high ATP consumption. In contrast, consistent with previous results [25], melatonin alone did not modify Complex V activity compared to controls (Figure 2I), whereas it counteracted CDDP‐induced activation. To our surprise, however, melatonin alone or combined with CDDP increased ATP levels in CAL 27/ABCB1 cells (Figure 2J), suggesting that melatonin inhibited the use of ATP for drug efflux in MDR cells.

ATP synthesis relies on a proton gradient maintained by NADH oxidation through the electron transport chain [32]. To evaluate effects on this pathway, we assessed the NADH/NAD+ ratio and Complex I activity (Figure 2K,L). In CAL 27/ABCB1 cells, melatonin, CDDP, and their combination reduced the NADH/NAD⁺ ratio and increased Complex I activity, an effect not seen in parental cells. These results were associated with increased ATP production, but as noted, we also had found that melatonin did not modify Complex V activity (Figure 2I). Therefore, we hypothesized that melatonin could induced mtROS, which could in turn directly oxidize NADH to NAD^+^.

### 3.3 Melatonin Reduces Drug Resistance Through Increased Mtros

**Figure 3:**
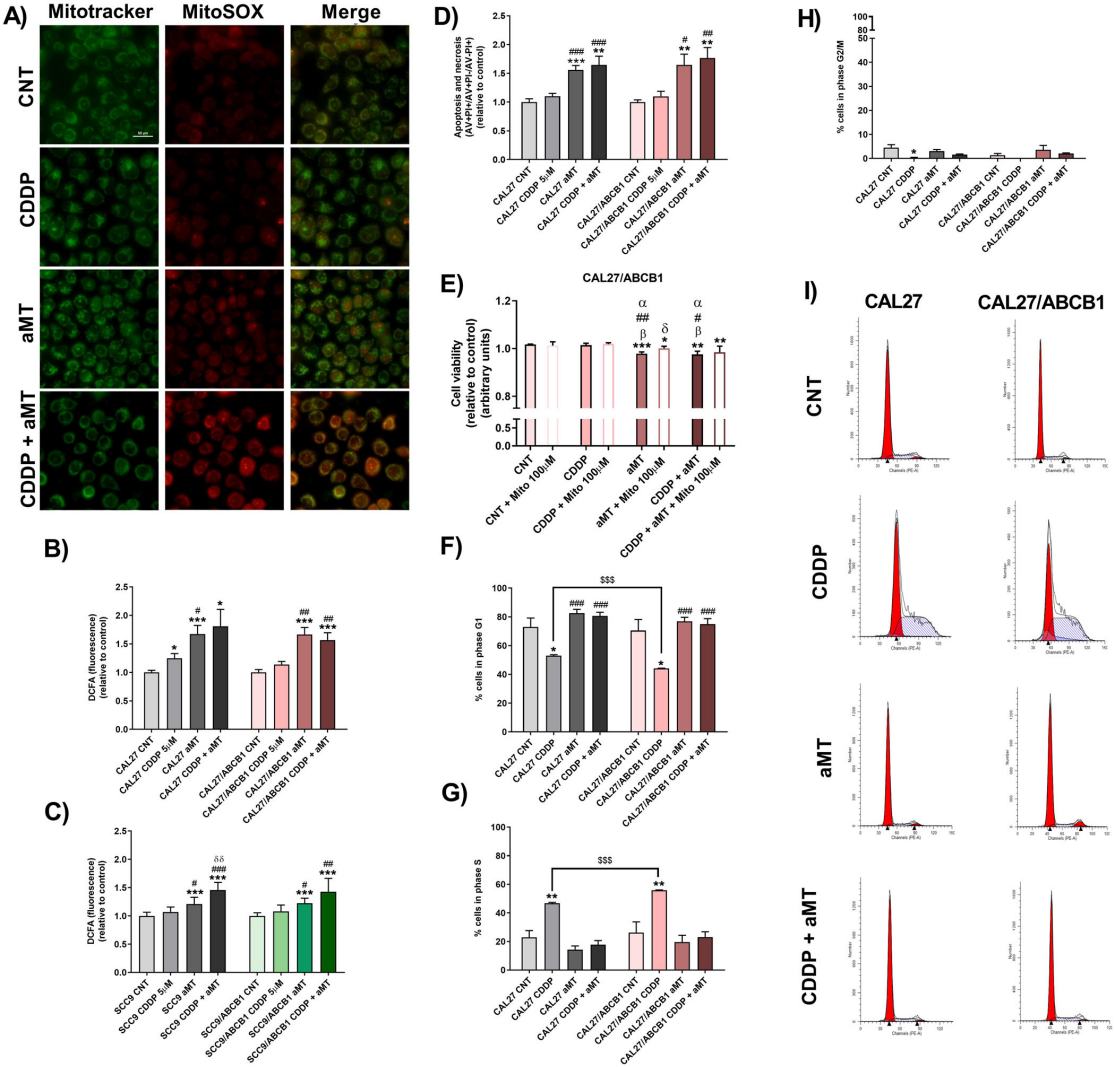
Melatonin increases ROS production and enhances the toxicity of CDDP in resistant cancer cells. (A) ROS (red) and mitochondria (green) detected by fluorescence microscopy after incubation with MitoSox‐Red (5 µM) and MitoTracker (50 nM) for 20 min in CAL27/ABCB1 cells. Scale bar = 50 µm. (B, C) Measurements of intracellular ROS levels by fluorimetry after staining with the DCFA fluorescent probe in CAL27/ABCB1, SCC9/ABCB1 and parental cells. (D). Apoptosis level (AV+ /PI+ and AV+ /PI−) analyzed by flow cytometry in CAL27/ABCB1 and parental cells. (E) Cell viability percentage after pretreatment with mitochondrial antioxidant MitoTEMPO during 1 h at 100 µM. (F–H) Percentage of cells in each cell cycle phase in CAL27/ABCB1 and parental cells (I) and representative plots showing cell redistribution. Treatements include vehicle (control), melatonin (aMT) at 1000 µM for 42 h, cisplatin (CDDP) at 5 µM for 18 h, or combined treatment. Data are presented as mean ± standard error of the mean (*n* = 3–12) for each group; one‐tailed unpaired t‐test; *p < 0.05; **p < 0.01; ***p < 0.001 versus control; ^#^ *p* < 0.05; ^##^ *p* < 0.01; ^###^ *p* < 0.001 versus cddp group; ^δ^ *p* < 0.05; ^δδ^ *p* < 0.01; ^$^ *p* < 0.05; ^$$^ *p* < 0.01; ^$$$^ *p* < 0.001; α*p* < 0.05; β*p* < 0.05.

Given our previous findings that melatonin induces tumor cell apoptosis through excessive ROS production [25], we addressed whether it also induces ROS in HNSCC/ABCB1 cells. Compared to the control group, melatonin alone or in combination with CDDP increased ROS levels in both parental and ABCB1‐overexpressing CAL‐27 and SCC9 cells (Figure 3A–C). Of note, ROS produced by melatonin exposure were clearly colocalized with the mitochondria (Figure 3A), whereas ROS production induced by CDDP alone was minimal (Figure 3A–C).

Melatonin‐induced ROS would be predicted to oxidize NADH, reducing the mitochondrial proton gradient. Flattening the gradient would in turn disrupt ATP synthesis and lead to efflux pump dysfunction because of insufficient energy. Therefore, an increase in mtROS would be expected overcome chemoresistance to CDDP. We found that melatonin alone or combined with CDDP significantly enhanced apoptosis compared to the control in both parental and CDDP‐resistant CAL 27/ABCB1 cells (Figure 3D). To further confirm the mitochondrial origin of melatonin‐induced ROS in resistant cancer cells, we pretreated the cells with MitoTEMPO, a mitochondria‐targeted antioxidant, and subsequently assessed cell viability. Consistent with our hypothesis, MitoTEMPO markedly attenuated melatonin‐induced cell death, indicating that the cytotoxic effect of melatonin is largely mediated by mtROS. In contrast, the cytotoxic activity of CDDP was unaffected by MitoTEMPO pretreatment (Figure 3E).

Given that excessive ROS may cause cell death and cell cycle arrest [33], we next analyzed cell cycle progression. In both parental and MDR cells, melatonin alone or in combination with CDDP increased the percentage of cells in G1 (Figure 3F,G) and decreased cell proliferation in S phase compared to CDDP treatment alone (Figure 3G,I). However, compared to parental cells, CAL‐27/ABCB1 cells showed higher proliferation in S phase after CDDP treatment (Figure 3G,I). Greater arrest in G2/M following CDDP treatment was observed in sensitive cells compared with resistant CAL‐27/ABCB1 cells. No statistical differences in the percentage of cells in G2/M were observed with melatonin treatment (Figure 3H). Taken together, these findings suggest that targeting mitochondrial ROS with a combination of melatonin and CDDP may overcome MDR in resistant cells.

### 3.4 Melatonin Alters Mitochondrial Morphology in CAL‐27/ABCB1 Xenografts

To explore melatonin′s mitochondrial effects on CDDP resistance in vivo, we examined tumor ultrastructure via transmission electron microscopy (TEM) in mouse xenograft models using CAL 27 and CAL 27/ABCB1 cells. Mice received intratumoral melatonin (3% daily) and weekly intraperitoneal CDDP for 35 days.

**Figure 4:**
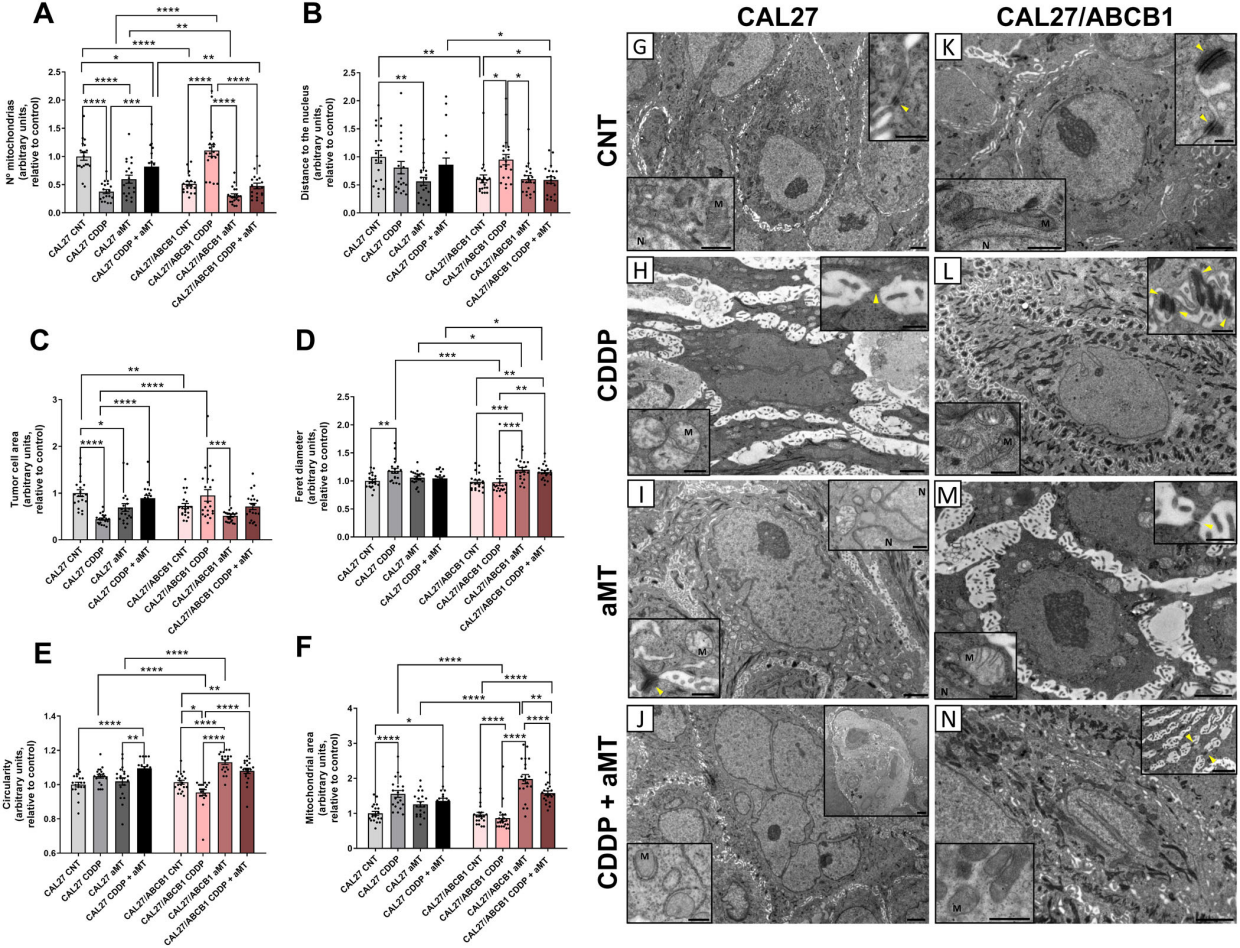
Intratumoral administration of melatonin modifies mitochondrial morphology in CAL 27/ABCB1 xenografts. (A–E) Quantification in transmission electron microscopy images of (A) the number of mitochondria per cell, (B) distance from the mitochondria to the nucleus, (C) tumor cell area, (D) Feret′s diameter of the mitochondria, (E) mitochondrial circularity, and (F) mitochondrial area. (G–J) Representative transmission electron microscopy images of CAL 27 cells from tumors with (G) vehicle treatment (CNT) (inserts: mitochondria [left] and a junction complex [right, yellow arrowhead]); (H) CDDP treatment (inserts: two mitochondria [left] and a junction complex [yellow arrowhead, right]); (I) aMT treatment (inserts: mitochondrion, dilated rough endoplasmic reticulum, and junction complex [yellow arrowhead, left] and mitochondrion, lipid droplet, and nuclear invagination [right]); and (J) CDDP and aMT treatment combined (inserts: mitochondria surrounded by rough endoplasmic reticulum [left] and cell death debris zone [right]). (K–N) Representative transmission electron microscopy images of CAL 27/ABCB1 cells from tumors with (K) vehicle treatment (CNT) (inserts: mitochondria [left] and a junction complex [yellow arrowhead, right]); (L) CDDP treatment (inserts: mitochondria [left] and many junction complexes [yellow arrowheads, right]); (M) aMT treatment (inserts: a mitochondrion [left] and a junction complex [yellow arrowhead, right]); and (N) CDDP and aMT combined treatment (inserts: some mitochondria [left] and junction complexes between cytoplasmic extensions with plenty of intercellular spaces [yellow arrowheads, right]). Scale bars: 2 µm, panoramic; 500 nm, inserts. Data are means ± standard error of the mean (*n* = 20 cells for each group). One‐tailed unpaired t‐test: *p < 0.05; **p < 0.01; ***p < 0.001 ****p < 0.0001. *N* = nucleus. *M* = mitochondria.

First, the morphological changes associated with ABCB1 overexpression were confirmed. ABCB1 overexpression in grafted mice was associated with fewer mitochondria (Figure 4A), and a perinuclear localization (Figure 4B). However, CDDP treatment in CAL 27/ABCB1 cells (Figure 4L) increased mitochondrial number (Figure 4A), reduced circularity (Figure 4E), and promoted cytoplasmic distribution (Figure 4B). Additionally, these CAL 27/ABCB1 cells contained a greater amount of keratin and numerous well‐developed junctional complexes (Figure 4L, top right). In contrast, CDDP exposure in the parental cells (Figure 4H) resulted in clear signs of cell damage and death, including pyknotic nuclei, heterogeneous dense bodies, and an increase in intercellular spaces, along with a notable reduction in cell size (Figure 4C) from cytoplasmic contraction caused by the loss of cytoplasmic components and organelles. Only small amounts of keratin were detected, and fewer microvilli with poorly developed junctional complexes were observed by comparison to control cells (Figure 4H, top right). These CDDP‐exposed cells also had fewer mitochondria (Figure 4A), which were highly altered, larger (Figure 4D,F), circular (Figure 4E), and empty because of the loss of cristae (Figure 4H, lower left). Overall, these findings suggest that CAL 27/ABCB1 cells remained in an active state to resist CDDP treatment.

In CAL 27/ABCB1 xenografts treated with melatonin (Figure 4M), tumor cells exhibited a morphology completely different from those treated with CDDP (Figure 4L). In the melatonin‐exposed tumors, cells displayed features of cellular damage, including increased intercellular spaces due to a reduction in cell size (Figure 4C), sparse microvilli with few junctional complexes (Figure 4M, top right), low keratin levels, and a dilated rough endoplasmic reticulum.

In CAL 27/ABCB1 cells, melatonin alone (Figure 4M) or with CDDP (Figure 4N) reduced mitochondrial numbers (Figure 4A), increased their circularity (Figure 4E), and promoted perinuclear accumulation (Figure 4E), potentially limiting ATP supply to efflux pumps in the plasma membrane [32]. Finally, combined CDDP and melatonin treatment induced nuclear invaginations and signs of cell damage, including increased intercellular spaces (Figure 4N, top right) and cell death (Figure 4J, top right) in both ABCB1 and parental cells.

### 3.5 Melatonin Overcomes CDDP Resistance In vivo

**Figure 5:**
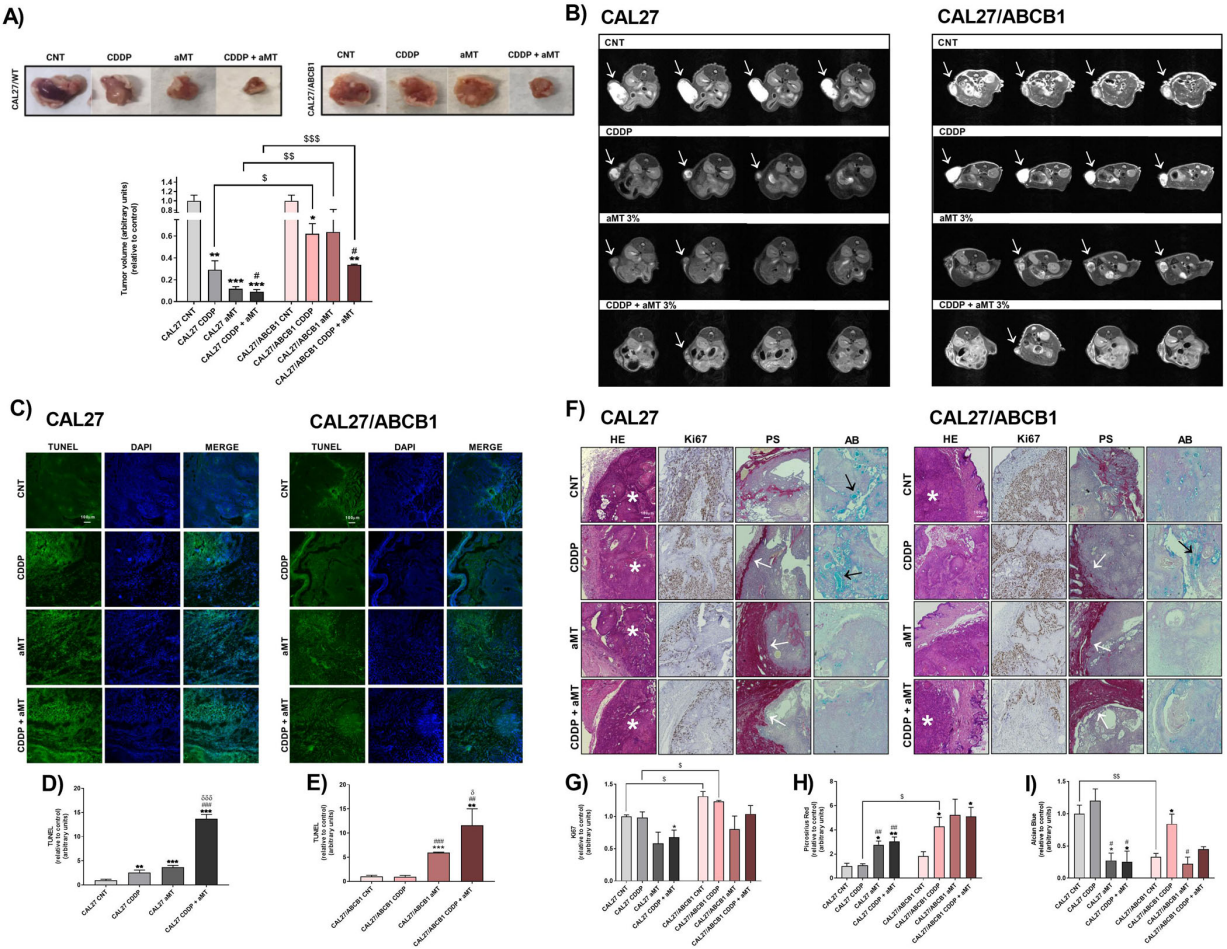
Intratumoral administration of melatonin overcomes CDDP resistance in CAL 27/ABCB1 xenografts. (A) Tumor volume after 35 days of treatment for mice bearing CAL 27 and CAL 27/ABCB1 tumors. (B) Representative axial T2‐weighted images obtained by MRI of mice bearing CAL 27 and CAL 27/ABCB1 tumors. White arrows indicate tumor mass. (C–E) TUNEL+ nuclei (apoptotic nuclei, green) and DAPI‐stained nuclei (total nuclei, blue) of the corresponding zone of the analyzed tumor in CAL 27 and CAL 27/ABCB1 tumors. Scale bar = 100 µm. (F‐I) Histological analysis of hematoxylin–eosin‐stained CAL 27 and CAL 27/ABCB1 tumors. Collagen capsule, Picrosirius red staining; mucin glandular structures stained by Alcian blue. Immunohistochemical identification of proliferating cells with Ki‐67 antibody (brown). Scale bar = 100 µm. Black arrows indicate the capsule stained by Picrosirius red. Experimental groups include vehicle (control), cisplatin at 4 mg/kg day once per week (CDDP), intratumoral melatonin at 3% every day (aMT), and the combination of CDDP plus intratumoral melatonin at 3% (CDDP + aMT). Data are means ± standard error of the mean (*n* = 3–5 per group). One‐tailed unpaired t‐test: *p < 0.05; **p < 0.01; ***p < 0.001 versus control tumors; ^#^ *p* < 0.05; ^##^ *p* < 0.01; ^###^ *p* < 0.001 versus CDDP tumors; ^δ^ *p* < 0.05; ^δδ^ *p* < 0.01; ^δδδ^ *p* < 0.001 versus aMT tumors; ^$^ *p* < 0.05; ^$$^ *p* < 0.01; ^$$$^ *p* < 0.001.

As shown in Figure 5A, CDDP alone was less effective in inhibiting tumor growth in CAL 27/ABCB1 than in CAL 27 xenografts. Melatonin enhanced CDDP′s cytotoxicity, significantly reducing tumor volume in both models compared to controls. These results were confirmed by MRI (Figure 5B), which indicated practically no tumors in mice that received the combined treatment. Effectively, the TUNEL assay showed that melatonin enhanced CDDP toxicity by increasing apoptosis in cells overexpressing ABCB1 (Figure 5C–E). These results highlight the great clinical potential of melatonin as an adjuvant to overcome chemoresistance to CDDP.

In line with the above results, histology revealed a significant increase in the proliferation of ABCB1‐overexpressing resistant tumors compared with non‐resistant tumors (Figure 5F,G). Moreover, CDDP treatment resulted in a significant increase in the collagen‐rich capsule (Figure 5F,H) and adenosquamous areas in chemoresistant tumors (Figure 5F,I) compared to the control group, without any significant effect in non‐resistant tumors. In contrast, co‐treatment with intratumoral melatonin and CDDP reduced proliferation and adenosquamous areas (Figure 5F,G,I) and significantly increased the collagen‐rich capsule (Figure 5F,H) in both resistant and non‐resistant tumors. The increase in the collagen‐rich capsule and the reduction in adenosquamous areas have been associated with better cancer outcomes [27].

Taken together, these data indicate that intratumoral melatonin enhances CDDP cytotoxicity and supports reduced drug resistance. The findings suggest the potential of melatonin to improve the effectiveness of existing anticancer therapies.

## 4 Discussion

Chemoresistance is a major cause of therapeutic failure and high mortality rates in HNSCC [34, 35]. The drug efflux pump ABCB1 plays a key role in promoting chemoresistance by actively effluxing a wide range of chemotherapeutic agents from tumor cells [36, 37, 38]. However, its widespread expression in healthy tissues [14] makes it a challenging target for selective therapy [38].

The impact of melatonin on MDR human cancer cells remains poorly understood, despite its potential therapeutic relevance, and research into its connection to MDR mechanisms has thus far been limited [39, 40, 41, 42]. To elucidate the molecular mechanisms by which melatonin sensitizes drug‐resistant cells to cancer therapy, we investigated its effects on ABCB1‐overexpressing cells. Our findings demonstrate for the first time that melatonin negatively regulates ABCB1 by modulating mitochondrial metabolism and redox balance, making it a promising candidate for combining with standard chemotherapy. Our current findings indicate that mitochondrial metabolism and redox balance are critical for the function of ABC transporters, facilitating drug efflux and driving chemoresistance in cancer cells. Taken together, these results are consistent with earlier reports demonstrating a link between intracellular ATP level and drug‐resistant cells [43, 44, 45, 46].

The current respiration analyses have revealed distinct metabolic profiles between resistant and non‐resistant cell lines. ABCB1‐overexpressing cells showed an increased oxygen consumption rate to meet the high ATP demand. Melatonin combined with CDDP reduced electron transport system capacity in MDR cell lines, implying ongoing mitochondrial dysfunction. Consequently, the increase in mitochondrial disfunction could make cancer cells more sensitive to chemotherapy drugs [8]. Of note, in CAL 27/ABCB1 cells, melatonin alone or with CDDP increased Complex I activity (Figure 2L) as well as NADH consumption (Figure 2K). However, melatonin reduced complex V activity (Figure 2I) compared to CDDP alone. Although enhanced Complex I activity and a lower NADH/NAD⁺ ratio would typically be associated with increased ATP synthesis, the results instead revealed a rise in mtROS levels, induced by melatonin. The resulting accumulation of ROS ultimately triggered apoptosis, leading to cell death in cells overexpressing ABCB1. Importantly, the use of MitoTEMPO abrogated both ROS accumulation and the chemosensitizing effect of melatonin, providing direct evidence that mtROS generation is required for this process. These data highlight a novel role of melatonin as an ABCB1 inhibitor and are congruent with our previous finding demonstrating melatonin‐induced tumor cell apoptosis due to excessive ROS production [25].

These effects were associated with alterations in mitochondrial morphology in a mouse xenograft tumor model using CAL 27/ABCB1 cells. In CDDP‐treated CAL 27/ABCB1 cells, mitochondria were dispersed throughout the cytoplasm and less circular, whereas melatonin treatment led to fewer, larger, and more circular mitochondria with disorganized cristae clustered near the nucleus. This difference was probably the result of the ability of the CAL 27/ABCB1 cells to pump out CDDP. Given that efflux pumps are localized on the plasma membrane, perinuclear mitochondrial distribution may limit ATP supply to these pumps. This limitation could explain the elevated ATP levels in ABCB1 cells treated with melatonin alone or in combination with CDDP (Figure 2I and 2J), as the ATP may not have been consumed by the efflux pumps. It has been shown [18] that chemotherapeutics such as doxorubicin induce a redistribution of mitochondria from a perinuclear pattern to the cell periphery. This relocation places mitochondria close to the plasma membrane and transporters such as ABCB1, suggesting a spatial coupling between mitochondrial ATP supply and drug‐efflux activity. These changes likely contribute to chemotherapeutic‐induced, efflux‐mediated resistance. Moreover, a decrease in ATP production could result in insufficient energy to transport P‐gp efflux pumps to the plasma membrane [32]. The observed structural features of cellular deterioration suggest that ABCB1 overexpression contributed to CDDP resistance in tumor cells, whereas melatonin induced mitochondrial remodeling that may have contributed to overcoming this resistance. Therefore, we hypothesized that melatonin impaired ATP delivery to efflux pumps.

These effects of melatonin correlated with a marked inhibition of tumor growth. Intratumoral melatonin boosted the antitumor effect of CDDP, significantly reducing tumor volume and inducing extensive necrosis and apoptosis. Furthermore, treatment with melatonin was associated with a significant increase in collagen capsule formation and a reduction in adenosquamous areas, features that have been linked to improved cancer prognosis [27]. Of note, MRI analysis revealed complete tumor regression, further underscoring the therapeutic potential of melatonin as an adjuvant to enhance CDDP efficacy in chemoresistant tumors.

**Figure 6:**
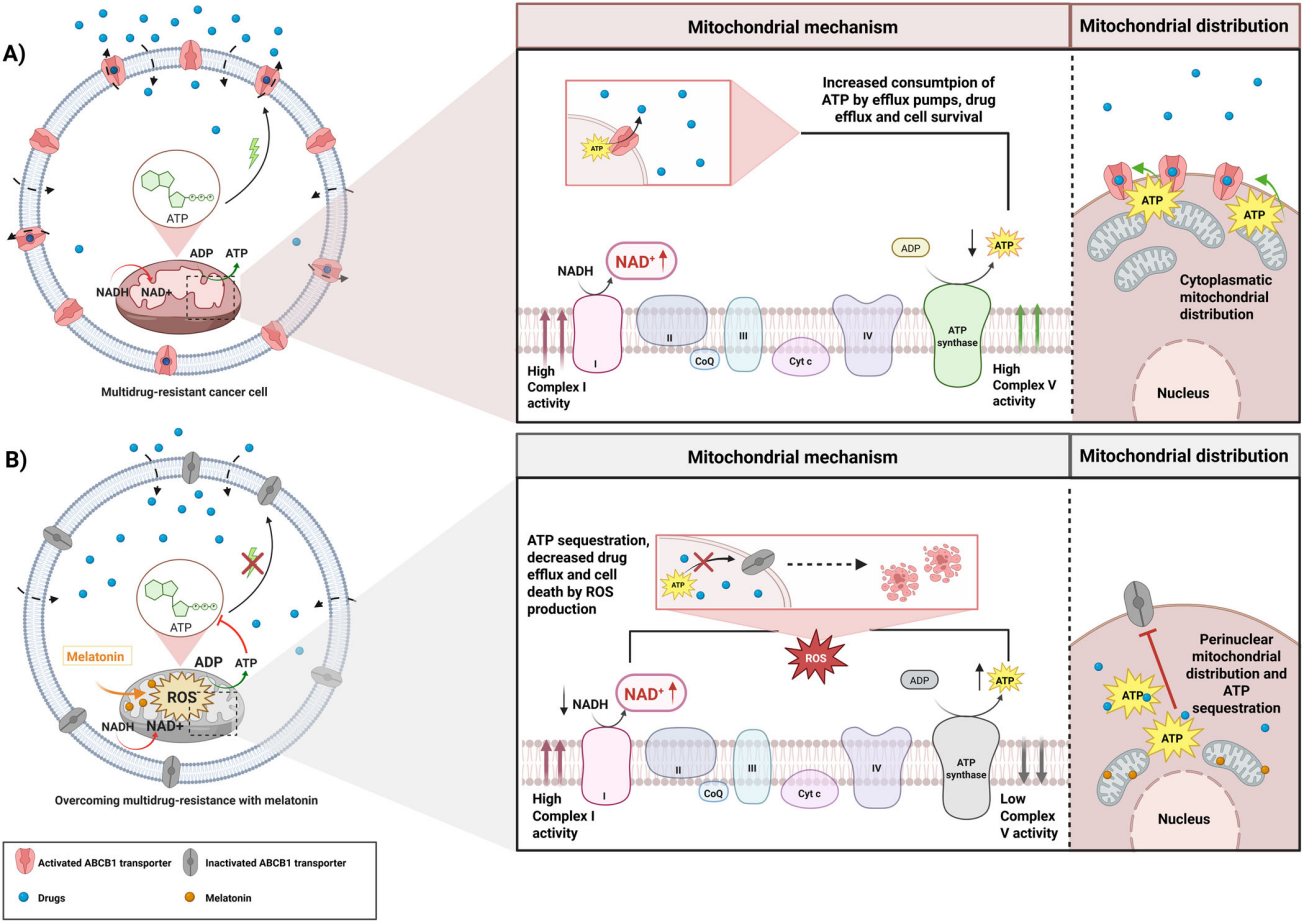
Melatonin overcomes ABCB1 drug resistance by boosting mitochondrial ROS and limiting ATP for efflux pumps. (A) Mitochondrial metabolism and distribution in MDR cancer cells without melatonin treatment. Treatment with CDDP or doxorubicin promotes NADH oxidation, leading to ATP production required for the activity of the ABCB1 efflux pump. Mitochondria are predominantly distributed throughout the cytoplasm. (B) Mitochondrial metabolism and distribution in MDR cancer cells treated with melatonin plus CDDP. Melatonin promotes Complex I activity and NADH oxidation but reduces complex V activity compared with CDDP, leading to increased ROS levels. Mitochondria are predominantly redistributed toward the perinuclear region, limiting ATP availability for the ABCB1 efflux pump membrane and decreasing its presence at the plasma.

In summary, the current results indicate that melatonin acts on mitochondria by generating ROS and promoting NADH oxidation, leading to reduced ATP production. Furthermore, it induces mitochondrial redistribution in the cell, potentially impairing ATP‐dependent efflux pump function and decreasing ABCB1 levels (see Figure 6 for an overview of pathways). The effect is compromised function of efflux pumps that overcomes the drug resistance of MDR ABCB1‐overexpressing cancer cells both in vitro and in vivo. These results suggest promising new targets for fighting MDR in cancer treatment.

## Author Contributions

Conceptualization, Germaine Escames; methodology, Germaine Escames, A.L‐R., Laura Martinez‐Ruiz, Javier Florido; formal analysis, Germaine Escames, A.L‐R., Laura Martinez‐Ruiz; investigation, A.L‐R., Laura Martinez‐Ruiz, R.M‐G., Javier Florido, F.B‐C., S‐T.A.,Y.R‐C., M.M‐E., P.G‐G., N.M‐P.; resources, Germaine Escames, J.M.G‐V., Víctor Carriel, Jean‐Francois Dumas, Francisco Martín, Christophe Vandier; writing – original draft preparation, Germaine Escames and A.L‐R.; writing – review and editing, Germaine Escames, A.L‐R., J.M.G‐V., Víctor Carriel, Jean‐Francois Dumas, Francisco Martín, Christophe Vandier, D.A‐C.; supervision, Germaine Escames, Víctor Carriel, Jean‐Francois Dumas, Francisco Martín, Christophe Vandier, Yang Yang, D.A‐C.; project administration, Germaine Escames; funding acquisition, Germaine Escames.

## Conflicts of Interest

The authors declare no conflicts of interest.

## Supporting information


**Table S1:** In vitro effects of CDDP, doxorubicin, melatonin, and their combined treatments on proliferation inhibition (%) in parental and drug‐resistant cell lines.

## Data Availability

The data that support the findings of this study are available from the corresponding author upon reasonable request.

## References

[1] M. M. Gottesman , T. Fojo , and S. E. Bates , “Multidrug Resistance in Cancer: Role of ATP‐Dependent Transporters,” Nature Reviews Cancer 2, no. 1 (2002): 48–58, 10.1038/NRC706. 11902585

[2] M. M. Gottesman and V. Ling , “The Molecular Basis of Multidrug Resistance in Cancer: The Early Years of P‐Glycoprotein Research,” FEBS Letters 580, no. 4 (2006): 998–1009, 10.1016/J.FEBSLET.2005.12.060. 16405967

[3] Z. Országhová , K. Kalavska , M. Mego , and M. Chovanec , “Overcoming Chemotherapy Resistance in Germ Cell Tumors,” Biomedicines 10, no. 5 (2022): 972, 10.3390/biomedicines10050972. 35625709 PMC9139090

[4] K. Bukowski , M. Kciuk , and R. Kontek , “Mechanisms of Multidrug Resistance in Cancer Chemotherapy,” International Journal of Molecular Sciences 21, no. 9 (2020): 3233, Bukowski K, Kciuk M, Kontek R. Mechanisms of multidrug resistance in cancer chemotherapy. Int J Mol Sci. 2020;21(9), 10.3390/ijms21093233. 32370233 PMC7247559

[5] Q. Yang , J. Xu , J. Gu , et al., “Extracellular Vesicles in Cancer Drug Resistance: Roles, Mechanisms, and Implications,” Advanced Science 9, no. 34 (2022): 1–33, 10.1002/advs.202201609. PMC9731723 36253096

[6] G. Szakács , J. K. Paterson , J. A. Ludwig , C. Booth‐Genthe , and M. M. Gottesman , “Targeting Multidrug Resistance in Cancer,” Nature Reviews Drug Discovery 5, no. 3 (2006): 219–234, 10.1038/nrd1984. 16518375

[7] Y. Xie , S. Feng , F. He , et al., “Down‐Regulating Nrf2 by Tangeretin Reverses Multiple Drug Resistance to Both Chemotherapy and Egfr Tyrosine Kinase Inhibitors in Lung Cancer,” Pharmacological Research 186, no. September (2022): 106514, 10.1016/j.phrs.2022.106514. 36252771

[8] H. Wang , J. M. Li , W. Wei , et al., “Regulation of Atp‐Binding Cassette Subfamily B Member 1 by Snail Contributes to Chemoresistance in Colorectal Cancer,” Cancer Science 111, no. 1 (2020): 84–97, 10.1111/CAS.14253. 31774615 PMC6942434

[9] P. P. Naik , S. Mukhopadhyay , P. K. Panda , et al., “Autophagy Regulates Cisplatin‐Induced Stemness and Chemoresistance via the Upregulation of CD44, ABCB1 and ADAM17 in Oral Squamous Cell Carcinoma,” Cell Proliferation 51, no. 1 (2018): 12411, 10.1111/CPR.12411. PMC6528880 29171106

[10] Z. Li , C. Chen , L. Chen , et al., “STAT5a Confers Doxorubicin Resistance to Breast Cancer by Regulating Abcb1,” Frontiers in Oncology 11 (2021): 697950, 10.3389/FONC.2021.697950/BIBTEX. 34336684 PMC8320598

[11] R. Bakadlag , G. Limniatis , G. Georges , and E. Georges , “The Anti‐Estrogen Receptor Drug, Tamoxifen, Is Selectively Lethal to P‐Glycoprotein‐Expressing Multidrug Resistant Tumor Cells,” BMC Cancer 23, no. 1 (2023): 24, 10.1186/S12885-022-10474-X/FIGURES/7. 36609245 PMC9824978

[12] X. D. Dong , M. Zhang , C. Y. Cai , et al., “Overexpression of ABCB1 Associated With the Resistance to the KRAS‐G12C Specific Inhibitor ARS‐1620 in Cancer Cells,” Frontiers in Pharmacology 13 (2022): 843829, 10.3389/FPHAR.2022.843829. 35281897 PMC8905313

[13] M. B. Duz and O. F. Karatas , “Differential Expression of ABCB1, ABCG2, and KLF4 as Putative Indicators for Paclitaxel Resistance in Human Epithelial Type 2 Cells,” Molecular Biology Reports 48, no. 2 (2021): 1393–1400, 10.1007/S11033-021-06167-6/FIGURES/5. 33506275

[14] F. S. Chung , J. S. Santiago , M. F. Jesus , C. V. Trinidad , and M. F. See , “Disrupting P‐Glycoprotein Function in Clinical Settings: What Can We Learn From the Fundamental Aspects of This Transporter?,” American Journal of Cancer Research 6, no. 8 (2016): 1583–1598, accessed March 26, 2025, https://pmc.ncbi.nlm.nih.gov/articles/PMC5004065/. 27648351 PMC5004065

[15] I. Genovese , M. Carinci , L. Modesti , G. Aguiari , P. Pinton , and C. Giorgi , “Mitochondria: Insights Into Crucial Features to Overcome Cancer Chemoresistance,” International Journal of Molecular Sciences 22, no. 9 (2021): 4770, 10.3390/ijms22094770. 33946271 PMC8124268

[16] P. Jin , J. Jiang , L. Zhou , et al. Mitochondrial Adaptation in Cancer Drug Resistance: Prevalence, Mechanisms, and Management. Vol 15. 2022, 10.1186/s13045-022-01313-4. PMC9290242 35851420

[17] Y. Fu , F. Ricciardiello , G. Yang , et al., “The Role of Mitochondria in the Chemoresistance of Pancreatic Cancer Cells,” Cells 10, no. 3 (2021): 497, 10.3390/cells10030497. 33669111 PMC7996512

[18] E. L. Giddings , D. P. Champagne , M. H. Wu , et al., “Mitochondrial ATP Fuels ABC Transporter‐Mediated Drug Efflux in Cancer Chemoresistance,” Nature Communications 12, no. 1 (2021 12:1. 2021): 2804, 10.1038/s41467-021-23071-6. PMC8121950 33990571

[19] J. T. Hagen , M. M. Montgomery , E. M. Biagioni , et al., “Intrinsic Adaptations in Oxphos Power Output and Reduced Tumorigenicity Characterize Doxorubicin Resistant Ovarian Cancer Cells,” Biochimica et Biophysica Acta (BBA)—Bioenergetics 1863, no. 8 (2022): 148915, 10.1016/J.BBABIO.2022.148915. 36058252 PMC9661894

[20] M. Jadhao , E. M. Tsai , H. C. Yang , et al., “The Long‐Term Dehp Exposure Confers Multidrug Resistance of Triple‐Negative Breast Cancer Cells Through Abc Transporters and Intracellular Ros,” Antioxidants 10, no. 6 (2021): 949, 10.3390/antiox10060949. 34208283 PMC8230873

[21] E. Panieri , A. Buha , P. Telkoparan‐akillilar , et al., “Potential Applications of NRF2 Modulators in Cancer Therapy,” Antioxidants 9, no. 3 (2020): 193, 10.3390/antiox9030193. 32106613 PMC7139512

[22] A. Cort , T. Ozben , L. Saso , C. De Luca , and L. Korkina , “Redox Control of Multidrug Resistance and Its Possible Modulation by Antioxidants,” Oxidative Medicine and Cellular Longevity 2016 (2016): 4251912, 10.1155/2016/4251912. 26881027 PMC4736404

[23] P. Dauer , N. S. Sharma , V. K. Gupta , et al., “GRP78‐mediated Antioxidant Response and Abc Transporter Activity Confers Chemoresistance to Pancreatic Cancer Cells,” Molecular Oncology 12, no. 9 (2018): 1498–1512, 10.1002/1878-0261.12322. 29738634 PMC6120253

[24] A. Guerra‐Librero , B. I. Fernandez‐Gil , J. Florido , et al., “Melatonin Targets Metabolism in Head and Neck Cancer Cells by Regulating Mitochondrial Structure and Function,” Antioxidants 10, no. 4 (2021): 603, 10.3390/antiox10040603. 33919790 PMC8070770

[25] J. Florido , L. Martinez‐Ruiz , C. Rodriguez‐Santana , et al., “Melatonin Drives Apoptosis in Head and Neck Cancer by Increasing Mitochondrial ROS Generated via Reverse Electron Transport,” Journal of Pineal Research 73, no. 3 (2022): 1–15, 10.1111/jpi.12824. PMC9541246 35986493

[26] B. I. Fernandez‐Gil , A. Guerra‐Librero , Y. Q. Shen , et al., “Melatonin Enhances Cisplatin and Radiation Cytotoxicity in Head and Neck Squamous Cell Carcinoma by Stimulating Mitochondrial ROS Generation, Apoptosis, and Autophagy,” Oxidative Medicine and Cellular Longevity 2019 (2019): 1–12, 10.1155/2019/7187128. PMC6421819 30944696

[27] L. Martinez‐Ruiz , J. Florido , C. Rodriguez‐Santana , et al., “Intratumoral Injection of Melatonin Enhances Tumor Regression in Cell Line‐Derived and Patient‐Derived Xenografts of Head and Neck Cancer by Increasing Mitochondrial Oxidative Stress,” Biomedicine & Pharmacotherapy = Biomedecine & Pharmacotherapie 167, no. July (2023): 115518, 10.1016/j.biopha.2023.115518. 37717534

[28] J. Florido , C. Rodriguez‐Santana , L. Martinez‐Ruiz , et al., “Understanding the Mechanism of Action of Melatonin, Which Induces Ros Production in Cancer Cells,” Antioxidants 11, no. 8 (2022): 1621, 10.3390/antiox11081621. 36009340 PMC9404709

[29] C. R. Fairchild , S. P. Ivy , C. S. Kao‐Shan , et al., “Isolation of Amplified and Overexpressed DNA Sequences From Adriamycin‐Resistant Human Breast Cancer Cells,” Cancer Research 47, no. 19 (1987): 5141–5148. 2441861

[30] M. E. Bosch , A. J. R. Sánchez , F. S. Rojas , and C. B. Ojeda , “Analytical Methodologies for the Determination of Cisplatin,” Journal of Pharmaceutical and Biomedical Analysis 47, no. 3 (2008): 451–459, 10.1016/j.jpba.2008.01.047. 18343619

[31] Y. Q. Shen , A. Guerra‐Librero , B. I. Fernandez‐Gil , et al., “Combination of Melatonin and Rapamycin for Head and Neck Cancer Therapy: Suppression of Akt/mTOR Pathway Activation, and Activation of Mitophagy and Apoptosis via Mitochondrial Function Regulation,” Journal of Pineal Research 64, no. 3 (2018): e12461, 10.1111/JPI.12461. 29247557

[32] H. Wang , Z. Gao , X. Liu , et al., “Targeted Production of Reactive Oxygen Species in Mitochondria to Overcome Cancer Drug Resistance,” Nature Communications 9, no. 1 (2018): 562, 10.1038/S41467-018-02915-8. PMC5805731 29422620

[33] B. Perillo , M. Di Donato , A. Pezone , et al., “Ros in Cancer Therapy: The Bright Side of the Moon,” Experimental & Molecular Medicine 52, no. 2 (2020 52:2. 2020): 192–203, 10.1038/s12276-020-0384-2. 32060354 PMC7062874

[34] S. Siemer , T. Fauth , P. Scholz , et al., “Profiling Cisplatin Resistance in Head and Neck Cancer: A Critical Role of the Vrac Ion Channel for Chemoresistance,” Cancers 13, no. 19 (2021): 4831, 10.3390/CANCERS13194831/S1. 34638315 PMC8508519

[35] Y. Garcia‐Mayea , C. Mir , L. Carballo , et al., “TSPAN1: A Novel Protein Involved in Head and Neck Squamous Cell Carcinoma Chemoresistance,” Cancers 12, no. 11 (2020): 3269, 10.3390/CANCERS12113269. 33167355 PMC7694336

[36] Y. Zu , Z. Yang , S. Tang , Y. Han , and J. Ma , “Effects of P‐Glycoprotein and Its Inhibitors on Apoptosis in K562 Cells,” Molecules 19, no. 9 (2014): 13061–13075, 10.3390/MOLECULES190913061. 25157469 PMC6270982

[37] R. Callaghan , F. Luk , and M. Bebawy , “Inhibition of the Multidrug Resistance P‐Glycoprotein: Time for a Change of Strategy?,” Drug Metabolism and Disposition 42, no. 4 (2014): 623–631, 10.1124/DMD.113.056176. 24492893 PMC3965902

[38] N. Fultang , A. Illendula , J. Lin , M. K. Pandey , Z. Klase , and B. Peethambaran , “ROR1 Regulates Chemoresistance in Breast Cancer via Modulation of Drug Efflux Pump Abcb1,” Scientific Reports 10, no. 1 (2020 10:1. 2020): 1821, 10.1038/s41598-020-58864-0. 32020017 PMC7000766

[39] M. Dehghanzad , M. Mohammadi , M. Nejati , et al., “The Potential Therapeutic Effect of Melatonin in Oxaliplatin Combination Therapy Against Chemoresistant Colorectal Cancer Cells,” Molecular Biology Reports 51, no. 1 (2024): 348, 10.1007/S11033-024-09316-9. 38401018

[40] A. Sakatani , F. Sonohara , and A. Goel , “Melatonin‐Mediated Downregulation of Thymidylate Synthase as a Novel Mechanism for Overcoming 5‐fluorouracil Associated Chemoresistance in Colorectal Cancer Cells,” Carcinogenesis 40, no. 3 (2018): 422–431, 10.1093/CARCIN/BGY186. PMC6514450 30590435

[41] R. Pariente , J. A. Pariente , A. B. Rodríguez , and J. Espino , “Melatonin Sensitizes Human Cervical Cancer HeLa Cells to Cisplatin‐Induced Cytotoxicity and Apoptosis: Effects on Oxidative Stress and DNA Fragmentation,” Journal of Pineal Research 60, no. 1 (2016): 55–64, 10.1111/JPI.12288. 26462739

[42] L. Chen , T. Sun , Y. Lv , et al., “Efficacy, Mechanism, and Safety of Melatonin‐Loaded on Thermosensitive Nanogels for Rabbit VX2 Tumor Embolization: A Novel Design,” Journal of Pineal Research 75, no. 3 (2023): e12900, 10.1111/JPI.12900. 37492880

[43] B. Verhalen , R. Dastvan , S. Thangapandian , et al., “Energy Transduction and Alternating Access of the Mammalian Abc Transporter P‐Glycoprotein,” Nature 543, no. 7647 (2017 543:7647. 2017): 738–741, 10.1038/nature21414. 28289287 PMC5558441

[44] A. S. Crystal , A. T. Shaw , L. V. Sequist , et al., “Patient‐Derived Models of Acquired Resistance Can Identify Effective Drug Combinations for Cancer,” Science 346, no. 6216 (2014): 1480–1486, 10.1126/SCIENCE.1254721. 25394791 PMC4388482

[45] H. Yang , Y. H. Geng , P. Wang , H. Q. Zhang , W. G. Fang , and X. X. Tian , “Extracellular ATP Promotes Breast Cancer Chemoresistance via HIF‐1α Signaling,” Cell Death & Disease 13, no. 3 (2022): 199, 10.1038/S41419-022-04647-6. 35236823 PMC8891368

[46] J. Dartier , E. Lemaitre , I. Chourpa , et al., “ATP‐Dependent Activity and Mitochondrial Localization of Drug Efflux Pumps in Doxorubicin‐Resistant Breast Cancer Cells,” Biochimica et Biophysica Acta (BBA)—General Subjects 1861, no. 5 (2017): 1075–1084, 10.1016/j.bbagen.2017.02.019. 28214549

