# Supplementary material for: Melatonin Overcomes Cancer Multidrug Resistance by Downregulating ABCB1 Expression and Modulating Mitochondrial Function
Source: J Pineal Res. 2025 Nov 4;77(6):e70096. doi: 10.1111/jpi.70096 (PMC12586911; doi:10.1111/jpi.70096)
Supplement: Supplementary file 1 — Table S1: In vitro effects of CDDP, doxorubicin, melatonin, and their combined treatments on proliferation inhibition (%) in parental and drug‐resistant cell lines. [file JPI-77-e70096-s001.docx]

**Supplementary material**

1. **MATERIALS AND METHODS**
   1. **Cell culture and treatment**

MCF7 cells were cultured in high-glucose Dulbecco’s Modified Eagle Medium (DMEM) supplemented with GlutaMAX™ (41965039, ThermoFisher Scientific, Waltham, MA, USA), 10% fetal bovine serum (FBS; 16000044, ThermoFisher Scientific), and 2% antibiotic/antimycotic solution (15240062, ThermoFisher Scientific). Cells were maintained at 37°C in a humidified atmosphere with 5% CO₂. Mycoplasma contamination was ruled out by PCR-based detection using a commercial kit (25235, LiliF Diagnostics, Burlington, MA, USA) according to the manufacturer’s instructions.

Melatonin (33457–24, Fagron Ibérica S.A.U., Terrassa, Spain) was dissolved in a 15% propane-1,2-diol (PG; 24414.296, VWR, Radnor, PA, USA) solution prepared in Dulbecco’s phosphate-buffered saline (DPBS; 14190094, Life Technologies, Carlsbad, CA, USA) to generate a stock solution. Cisplatin (CDDP; 1134357, Sigma, Madrid, Spain) and doxorubicin (D1515, Sigma) stock solutions were prepared according to standard procedures. Cells were treated with 1000 µM melatonin for 42 hours, 5 µM CDDP, or 0.1 µM doxorubicin for 18 hours. Control groups received vehicle treatment.

- 1. **Western blot analysis**

40 µg of denatured protein per sample were resolved on a 7.5% acrylamide/bis-acrylamide gels and subsequently transferred to a PVDF membrane (IPVH00010, Sigma). Membranes were blocked with 5% milk in PBS containing 0.1% Tween-20 and incubated with the primary antibody overnight at 4 °C. The primary antibody dilutions were as follows: Nrf2, 1:500 (PA5-27882, ThermoFisher Scientific) and MDR1/ABCB1, 1:500 (13131, Santa Cruz, Heidelberg, Germany). Immunoreactive bands were visualized using ECL™ Prime Western Blotting Detection Reagent (GE Healthcare Life Sciences, Barcelona, Spain) and quantified using ImageLab software after image acquisition with a ChemiDoc MP Imaging System (Bio-Rad). Protein expression levels were normalized to Vinculin at 1:500 (73614, Santa Cruz).

- 1. **Measurement of mitochondrial respiration**

Cells were seeded at a density of 8·10^4^ per well in DMEM in 24-well cell culture plates and allowed to adhere overnight. Prior to the assay, the medium was changed to base medium (102353-100, Seahorse Bioscience, Santa Clara, CA, USA) containing 10 mM glucose (D8375, Sigma), 5 mM sodium pyruvate, and 2 mM L-glutamine (25030081, ThermoFisher Scientific), and cells were equilibrated for 1 h at 37°C without CO_2_.

Basal OCR was measured and plotted as a function of cells under the basal condition, followed by the sequential addition of oligomycin 1 mM, an inhibitor of ATP synthase. Subsequently, two injections of the ionophore FCCP (C2920, Sigma) at 0.5 mM were performed (1 mM in total). Finally, rotenone/antimycin A (R8875, Sigma) (A8674, Sigma) (1 mM) was injected. Maximal mitochondrial electron transport system (ETS) capacity was measured after the addition of FCCP, and the residual nonmitochondrial OCR was assessed using rotenone/antimycin A. OCR values were normalized by protein content as measured by the Bradford assay.

- 1. **Electron transport chain (ETC) complex activity assays**

Mitochondrial respiratory chain activities were measured for complexes I and V using kits from Abcam according to the manufacturer’s instructions (ab109721 and ab109714, Abcam respectively, Cambridge, UK). Cell cultures were added to microplate wells that were precoated with capture antibodies specific for each mitochondrial respiratory chain complex. After the target was immobilized in the well, the complex activity was determined by following the oxidation and reduction of complex-specific substrates and a dye. The absorbance (450 and 340 nm, respectively) was measured at specific optical densities using a microplate reader spectrophotometer (Power Wave X-1; Bio-Tek Instruments, Inc., Winooski, VT, USA).

- 1. **Measurement of ROS production and mitochondrial mass**

Cells were incubated in complete medium with 5 μM MitoSox (M36008, ThermoFisher Scientific) and 50 nM MitoTracker (M7514, ThermoFisher Scientific) for 20 min at 37°C under 5% CO_2_ and washed with DPBS. Fluorescence was assessed by fluorescence microscopy (Nikon Eclipse Ni-U microscope).

ROS productions were measured using 100 µM 2’-7’-dichlorofluerescein diacetate (DCFH-DA, D6883, Sigma), which is transformed in the cell to fluorescent 2',7'-dichlorofluorescein (DCF). ROS levels were then measured with a microplate fluorescence reader FLx800 (Bio-Tek Instruments, Inc., Winooski, VT, USA) for 45 min every min at 485 nm to excitation and 530 nm to emission.

- 1. **Animal models and treatments**

Animals were maintained under controlled conditions (12:12 h light/dark cycle, 22 °C) with ad libitum access to standard chow and water. For xenograft implantation, CAL27 and CAL27/ABCB1 cell lines were utilized. Cell viability was confirmed through trypan blue exclusion. A cell suspension (4 x 10^6^ cells/0.2 mL) was subcutaneously injected into the left flanks of mice, embedded in a 1:1 mixture of DMEM without fenol red (31053028, ThermoFisher Scientific) and Matrigel Basement Membrane Matrix (VWR).

Body weight and tumor dimensions were monitored regularly. Tumor volume was calculated based on caliper measurements using the formula: volume = (width × length^2^)/2. Once the tumor volume reached 100-200 mm³ range, mice were randomly assigned to different treatment groups. Treatment included melatonin (aMT), CDDP, and a combination of CDDP and melatonin (CDDP + aMT), with a control group receiving vehicle treatment.

Melatonin was dissolved in propylene glycol (PG) and subsequently diluted to 37.5% in saline solution. CDDP was solubilized in saline solution at 1.2 mg/mL. Both drug formulations were sterilized through a 0.2 µm pore filter (#PN 4612, Pall Corporation LifeSciences, CA, USA) prior to administration.

*Melatonin was administered intratumorally every 24 hours for 35 days, using 3% concentration. The dosage was standardized at 60 µL of treatment solution per 100 mm3 of tumor volume.*

*For CDDP, the drug was administered intraperitoneally once per week at a dosaje of 4 mg/kg. When CDDP was combined with melatonin, melatonin was consistenly administered intratumorally every 24 hours at a 3% concentration, base on prior experimental findings*

- 1. **Magnetic Resonance Imaging**

Animals were anesthetized using isoflurane vaporized in oxygen. An induction concentration of 3.0% isoflurane was employed, followed by a maintenance concentration of 1.0-2.0%. Mice were positioned prone for transverse image acquisition covering the entire tumor volume. Image planning was based on sagittal and coronal scout views. A T2-weighted turbo spin-echo multi-slice sequence (T2W MS TSE) was implemented with the following parameters: field of view (FOV) 50 x 50 x 19 mm on the axial plane, 27 slices, voxel size 0.15 x 0.15 x 0.7 mm, TE of 97 ms, and repetition time (TR) 2800 ms.

- 1. **Electron microscopy (TEM)**

For electron microscopy, animals were subjected to intracardiac perfusion with 2% PFA-2% glutaraldehyde (A17876, ThermoFisher Scientific) in 0.1 M phosphate buffer (PB; pH 7.4). Then, tumors where removed and post-fixed with a solution of 2% osmium tetroxide in 0.1 M PB for 1h 30 min at room temperature, washed with deionized water and partially dehydrated in increasing concentrations of ethanol. Then, sections were contrasted with 2% uranyl acetate in 70% ethanol for 2 h 30 min at 4 °C, and, after that, dehydrated with 70%, 96%, 100% ethanol, and propylene oxide. Subsequently, samples were embedded in Durcupan epoxy resin (44611–44614, Sigma). After resin polymerization during 72 h at 70 ºC, a diamond knife was used to cut semithin sections (1.5 µm) using a UC7 ultramicrotome (Leica), which were stained with 1% toluidine blue for light microscopy. Once regions of the tumor core were selected for each sample, ultrathin sections (70–80 nm) were then cut, stained with lead citrate, and evaluated under a FEI Tecnai G2 Spirit transmission electron microscope (FEI Europe, Hillsboro, OR) equipped with a Xarosa digital camera (20 Megapixel resolution) using Radius image acquisition software (EMSIS GmbH, Münster, Germany).

- 1. **Histology**

Animals were euthanized via intracardiac perfusion with neutral buffered paraformaldehyde (3.7-4%). Following a 24-hour fixation period, tumors were longitudinally sectioned for histological processing. Tissue samples were dehydrated in ethanol, cleared in xylene, and embedded in paraffin. Deparaffinized and hydrated 5 µm tissue sections were subjected to histological staining.

Tumor encapsulation was quantified by calculating the tumor/capsule ratio on Picrosirius red-stained sections using ImageJ software. The percentage area occupied by mucopolysaccharides was determined similarly on Alcian Blue-stained sections. The proliferation index was calculated on Ki-67-stained sections counting the Ki-67 positive cells. For TUNEL assay, tumors were fixed in 4% PFA, cryopreserved in sucrose, and embedded in OCT. Nuclear staining was performed with Hoechst, and apoptotic cells were quantified by analyzing five random fields per slide under a fluorescence microscope.

1. **SUPPLEMENTARY FIGURES AND TABLES**

Figure S1


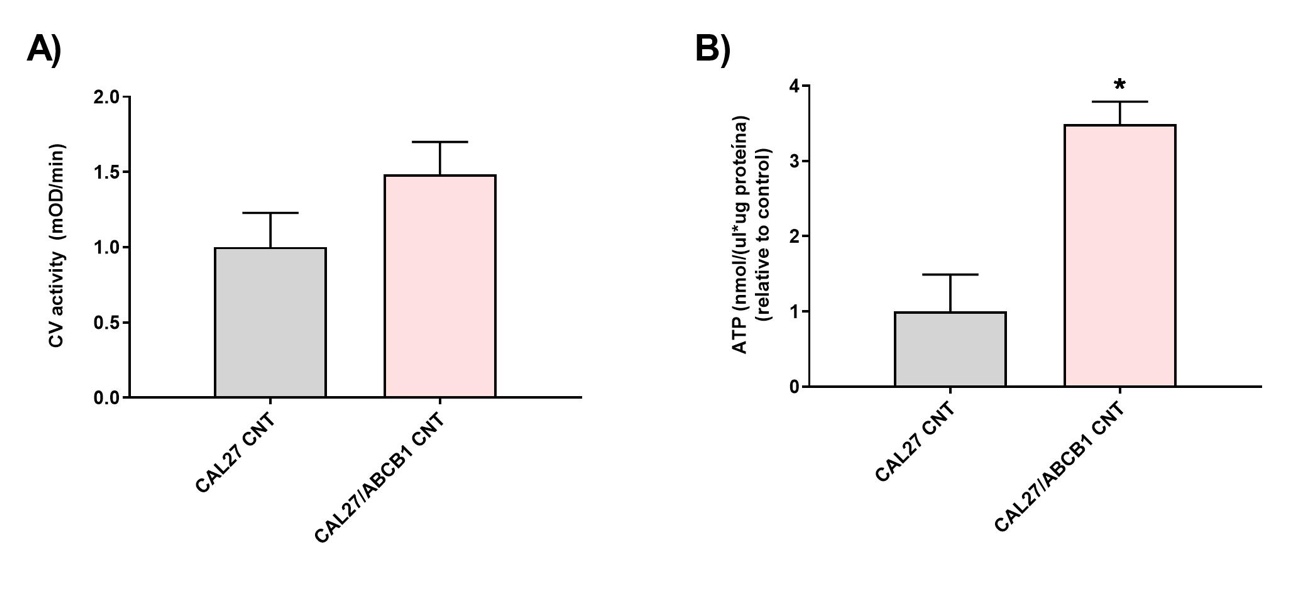


**Table S1:** In vitro effects of CDDP, doxorubicin, melatonin, and their combined treatments on proliferation inhibition (%) in parental and drug-resistant cell lines.

| Cell line %Cell proliferation inhibition | | | | | |
| --- | --- | --- | --- | --- | --- |
|  | **CDDP** | **DOX** | **aMT** | **CDDP + aMT** | **DOX + aMT** |
| **CAL27** | 19.4 | - | 14 | 26.2 | - |
| **CAL27/ABCB1** | 12 | - | 18.6 | 18 | - |
| **SCC9** | 28.2 | - | 24.3 | 46.2 | - |
| **SCC9/ABCB1** | 15.5 | - | 5.5 | 25.9 | - |
| **MCF7** | - | 45.6 | 57.6 | - | 77.8 |
| **MCF7/ABCB1** | - | 9.5 | 40.3 | - | 56.5 |
|  |  |  |  |  |  |

1. **LEGENDS FOR SUPPLEMENTARY FIGURES**

**Figure S1. The overexpression of ABCB1 impacts mitochondrial energy metabolism inCAL27 cell line.**

(A) Analysis of mitochondrial complex V activity by spectrophotometric analysis.

(B) ATP levels measured by fluorimetric test.

Data are presented as the means ± standard error of the mean (n = 3) for each group. One-tailed unpaired t-test: *p < 0.05
